# Mass testing—An underexplored strategy for COVID-19 control

**DOI:** 10.1016/j.xinn.2021.100114

**Published:** 2021-04-29

**Authors:** Mingwang Shen, Yanni Xiao, Guihua Zhuang, Yan Li, Lei Zhang

**Affiliations:** 1 China-Australia Joint Research Center for Infectious Diseases, School of Public Health, Xi'an Jiaotong University Health Science Center, Xi'an 710061, China; 2 School of Mathematics and Statistics, Xi'an Jiaotong University, Xi'an 710049, China; 3 Department of Population Health Science and Policy, Icahn School of Medicine at Mount Sinai, New York, NY 10029, USA; 4 Department of Obstetrics, Gynecology, and Reproductive Science, Icahn School of Medicine at Mount Sinai, New York, NY 10029, USA; 5 Melbourne Sexual Health Centre, Alfred Health, Melbourne, VIC, Australia; 6 Central Clinical School, Faculty of Medicine, Nursing and Health Sciences, Monash University, Melbourne, VIC, Australia; 7 Department of Epidemiology and Biostatistics, College of Public Health, Zhengzhou University, Zhengzhou 450001, China

## Main text

**Figure 1:**
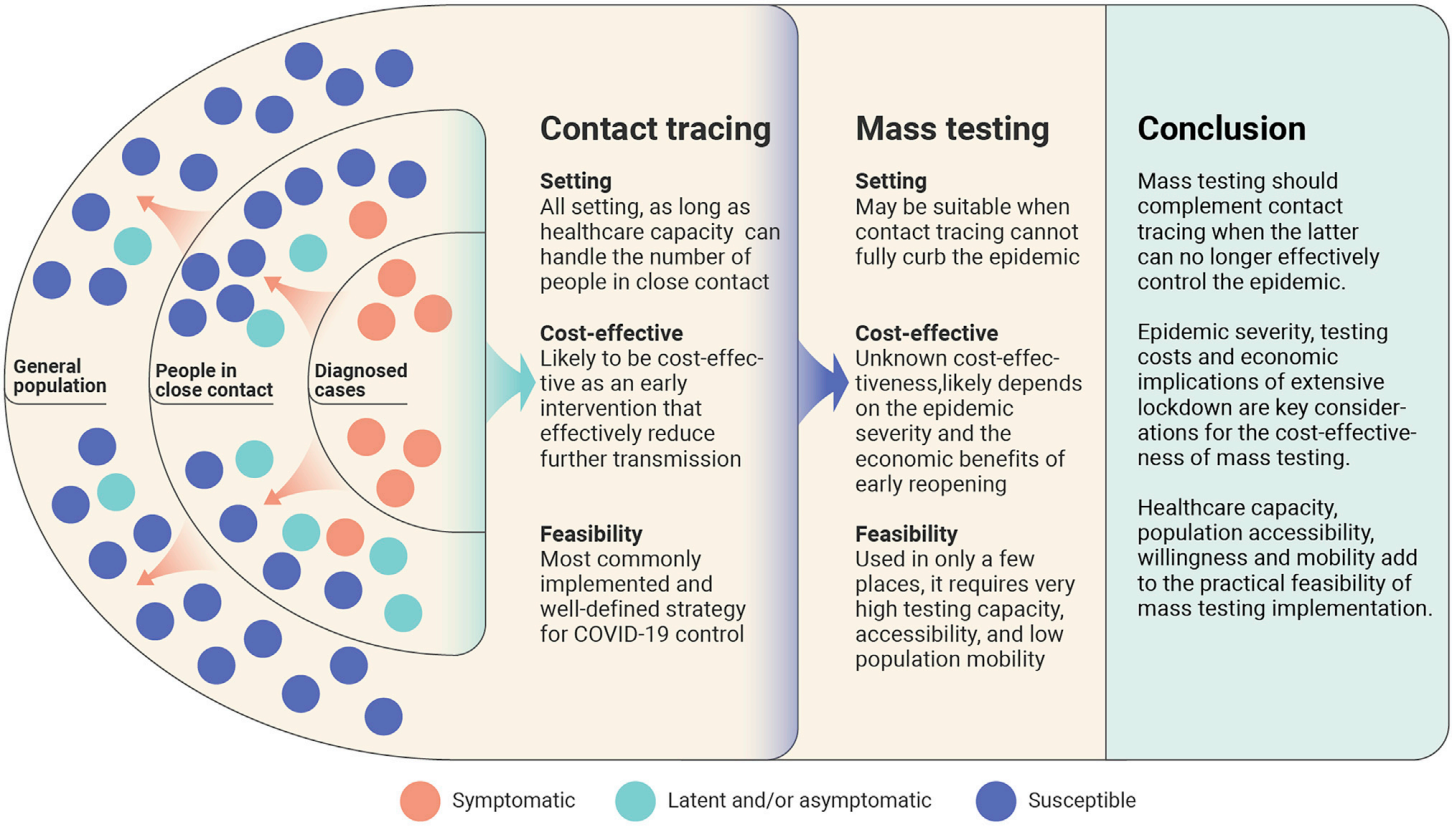
Diagram of controlling the transmission of COVID-19 with contact tracing and mass testing. Those susceptible could be infected by contacts with latent, asymptomatic, and undiagnosed symptomatic infections. Contact tracing is based on close contacts with daily new diagnosed symptomatic cases. Mass testing is implemented for the general population to timely identify more infections regardless of symptoms.

Mass testing is an intervention strategy for COVID-19 control in the general population regardless of the presentation of symptoms. It involves collecting nasal or pharyngeal swabs for DNA testing, often using the polymerase chain reaction method. Countries that have used mass testing consider it to be a viable strategy to control the COVID-19 pandemic as the strategy can potentially identify and isolate asymptomatic cases in the early stages of infection and reduce the risk of virus transmission. Since the reopening of Wuhan, China, in early April 2020, China has conducted mass testings in three megacities with populations of over 10 million, including Beijing and Qingdao^1^ at the beginning of their local outbreaks, and Wuhan immediately after the reopening.^2^ Several other smaller cities and districts, including Shijiazhuang, Dalian, Kashi, and Binhai New District in Tianjin, also conducted mass testing among their residents. In November 2020, after piloting mass testing in the city of Liverpool, England introduced mass testing in 67 local authorities in “tier 3” areas where community spread of COVID-19 was prevalent. Luxembourg and Slovakia also implemented similar mass testings at the national level to allow partial lockdown measures to be eased. Most previous decisions on mass testing were made on an ad hoc basis. Many questions about mass testing—such as the setting of implementation, the cost-effectiveness, and practical feasibility of mass testing—are yet to be answered. This study proposes a theoretical framework to address these issues and recommend potential solutions (Figure 1).

Mass testing should be implemented when contact tracing cannot effectively control the spread of the virus. A mathematical modeling study^3^ suggested that a new COVID-19 outbreak can be controlled within 3 months with highly effective contact tracing (tracing >80% infected individuals) and quarantine of infected individuals. This modeling projection has been confirmed by experiences in China during its first wave of the COVID-19 pandemic from February to April, 2020.^4^ The main purpose of Wuhan's post-reopening mass testing was to ensure the city was free from COVID-19 and rebuild the confidence of city residents in normal economic activities.^2^ In contrast, the purpose of subsequent mass testings in other Chinese cities was to rule out the risk of a potential large outbreak following the detection of a handful of cases. In theory, when community outbreaks are still in the early stages, an effective contact tracing may be sufficient to curb the epidemic, and a population-wide mass testing may not be needed. For most of the Chinese cities that implemented mass testing, the nearly 100% testing coverage provided important public health evidence that the vast majority of the infections, regardless of symptoms, were identified and subsequently isolated in the same way as contact tracing. Similarly, for many other countries that are still fighting an upward battle against the pandemic and contact tracing is insufficient to contain the spread of the virus, mass testing may provide a potential solution to curb the epidemic by identifying most infected individuals in a relatively short time.^5^ Mass testing is particularly helpful in identifying asymptomatic cases to prevent further transmission. However, until now, there is no consensus or common epidemiological indicator to guide nations or regions on the timing of shifting from contact tracing to mass testing. This will be largely dependent on the capacity of the healthcare system and feasibility implementation. Further future investigation is necessary to determine the appropriate threshold for initiating mass testing.

Mass testing needs to be cost-effective with its practical economic benefits outweighing its costs. First, when the epidemic in a population is severe, mass testing could detect a large number of otherwise undiagnosed cases and, hence, avert a large number of potential future infections. This would save sufficient healthcare costs to outweigh the investment in mass testing. Second, testing costs need to be sufficiently low. Batch testing (mix 5–10 samples into a “cluster” and test the cluster with a single test kit; further individual testing is required only if a positive cluster is detected) has been implemented in both China and Luxembourg, which proved to be an efficient way to test a large number of individuals in a short time for low-prevalence areas. However, the number of samples per batch is negatively associated with the prevalence of COVID-19 in the population. Smaller batches are necessary in high-prevalence areas. Repeated testing and confirmed diagnosis combined with clinical symptoms are needed to exclude the possible false-positive or false-negative cases due to imperfect sensitivity and specificity of the testing methods. Third, to minimize the economic disruption, mass testing may be implemented during a city lockdown when social contacts are at the lowest. Mass testing may help shorten the required duration of lockdowns and avert significant economic losses by enabling earlier reopening. Rigorous cost-effectiveness analyses from the perspectives of the health systems or the society are needed to investigate whether mass testing was cost-effective for the countries and regions that implemented this strategy.

Mass testing may be practically feasible under the following conditions. First, the testing capacity of a healthcare system determines its ability to organize and provide testing to a large population. Producing sufficient testing kits during a short period could be challenging, which would limit the testing capacity. Batch testing can improve testing capacity. An alternative way to cater for limited testing capacity is to test a relatively small proportion (e.g., 5%–10%) of the population in consecutive days and gradually build up the testing coverage over time.^5^ Second, to enable the best testing accessibility and population willingness, mass testing should be state-funded and provided to the population universally through primary care. The requirement of private or out-of-pocket payment may discourage socio-economically disadvantaged populations, especially those who are already being impacted by the unprecedented crisis. The testing service, including both commodity and personnel, should be made accessible to residential areas or workplaces to minimize traveling by and build-up of large crowds. The testing should be administered using an appointment system and distributed evenly among the general population, and strict sterilization is required to prevent potential transmission when a large number of people gather for mass testing. Third, practically, mass testing should not be repeatedly implemented as it itself is a significant interruption to the society. During its implementation in a city or suburb, the population mobility should be minimized with appropriate border closure or city lockdown to prevent the import of new cases from high-risk areas.

We conclude that mass testing should complement contact tracing when the latter can no longer effectively control the epidemic. Epidemic severity, testing costs, and economic implications of extensive lockdown are key considerations for the cost-effectiveness of mass testing. Healthcare capacity, population accessibility, willingness, and mobility add to the practical feasibility of mass testing implementation.
